# Rapid Determination of Nutmeg Shell Content in Ground Nutmeg Using FT-NIR Spectroscopy and Machine Learning

**DOI:** 10.3390/foods12152939

**Published:** 2023-08-02

**Authors:** Alissa Drees, Bernadette Bockmayr, Michael Bockmayr, Markus Fischer

**Affiliations:** 1 Hamburg School of Food Science, Institute of Food Chemistry, University of Hamburg, Grindelallee 117, 20146 Hamburg, Germany; alissa.drees@uni-hamburg.de; 2 Husarich GmbH, Peutestraße 53 D, 20539 Hamburg, Germany; b.bockmayr@husarich.com; 3 Department of Pediatric Hematology and Oncology, University Medical Center Hamburg-Eppendorf, Martinistraße 52, 20246 Hamburg, Germany; m.bockmayr@uke.de; 4 Research Institute Children’s Cancer Center Hamburg, Martinistr. 52, 20251 Hamburg, Germany; 5 Center for Hybrid Nanostructures (CHyN), Department of Physics, University of Hamburg, Luruper Chaussee 149, 22761 Hamburg, Germany

**Keywords:** near-infrared spectroscopy, food fraud, adulteration, authentication, SVM, chemometrics, *Myristica fragrans* Houtt

## Abstract

Nutmeg is a popular spice often used in ground form, which makes it highly susceptible to food fraud. Therefore, the aim of the present study was to detect adulteration of ground nutmeg with nutmeg shell via Fourier transform near-infrared (FT-NIR) spectroscopy. For this purpose, 36 authentic nutmeg samples and 10 nutmeg shell samples were analyzed pure and in mixtures with up to 50% shell content. The spectra plot as well as a principal component analysis showed a clear separation trend as a function of shell content. A support vector machine regression used for shell content prediction achieved an *R*^2^ of 0.944 in the range of 0–10%. The limit of detection of the prediction model was estimated to be 1.5% nutmeg shell. Based on random sub-sampling, the likelihood was found to be 2% that a pure nutmeg sample is predicted with a nutmeg shell content of >1%. The results confirm the suitability of FT-NIR spectroscopy for rapid detection and quantitation of the shell content in ground nutmeg.

## 1. Introduction

Nutmeg is a widely used spice that is known not only for its highly characteristic flavor but also for its various pharmacological effects [1,2,3]. It is made from the fruit of the nutmeg tree *Myristica fragrans* Houtt. The spice nutmeg is the dried kernel of the seed. This plant is indigenous to Indonesia, which remains the leading producer and exporter of nutmeg (>20,000 Mt/a) [4]. However, *Myristica fragrans* is now distributed in humid tropical and coastal regions worldwide. India is another major producer of nutmeg (~15,000 Mt/a) but almost exclusively for domestic consumption [4,5]. Nutmeg is mostly sold as whole fruit or ground and ready to use for cooking.

Compared to other food industry sectors, the spice industry is generally highly vulnerable to food fraud [6]. “Food Fraud” means an intentional adulteration of foodstuff for the purpose of increasing profits [7]. This was also recently emphasized by the results of an EU-wide coordinated control plan to determine the prevalence of fraudulent practices in the marketing of herbs and spices, which showed an overall rate of suspect samples of 17% [8]. The high vulnerability to intentional adulteration is mainly caused by the fact that spices are often marketed ground to be ready for use in compound foods. In the ground state, it is much more difficult to verify the authenticity of the food based on its morphology. Intentional adulteration with a low percentage usually cannot be detected based on sensory characteristics, and, therefore, analytical methods are required.

Nutmeg, as a high-priced spice mostly imported from Indonesia, is the subject of a comprehensive value-adding chain from harvesting to proper storage and thorough processing. A high-quality product is characterized by a valuable sensory profile, and as a top priority, efforts have to be made to avoid fungal infections and thus contamination with mycotoxins [9,10]. A general aspect that may encourage cases of food fraud is limited availability of the respective foodstuff. Higher transportation prices and longer transportation times, as seen during the last two years, contribute to a shortage of available spices that are imported from overseas, such as nutmeg [11,12]. These are reasons why nutmeg is at enhanced risk for adulteration with low-cost ingredients.

Consequently, nutmeg has been subject to falsification on several occasions in the past. For instance, in ground nutmeg, adulteration with coffee husks was reported in 2004 [13]. Moreover, nutmeg is suspected to be adulterated with various nutmeg by-products such as shell material (i.e., material of the seed coat), nutmeg pericarp, or spent powder (extracted nutmeg) without proper declaration [14,15]. Nutmeg shell is an easily available material during the harvest and processing of nutmeg that has sensory properties partly comparable to those of nutmeg. While nutmeg shell is a low-quality by-product of nutmeg and easily distinguishable from nutmeg in pure form, small amounts of added nutmeg shell in a ground nutmeg sample cannot be detected based on sensory tests. Although this underlines the urgent need for nutmeg authentication methods, few studies have been reported so far. The Codex Alimentarius does not include a specific acceptable value for extraneous matter such as nutmeg shell in ground nutmeg. However, an extraneous matter content of 0.5% is given for whole and broken nutmeg, which can be associated with a technically unavoidable amount [16].

Common analytical techniques used for food authentication include, for example, LC-MS, GC-MS, ICP-MS, and different spectroscopic methods [17,18,19,20,21,22]. To date, the adulteration of nutmeg with spent material has been studied with two different mass spectrometry methods [15,23]. In addition, DNA-based approaches were described by Tallei and Kolondam [24] and Swetha et al. [25] in order to differentiate *Myristica fragrans* from adulterants of another genus or species. Nonetheless, these methods are not suitable when adulteration is carried out with material from the same species, such as nutmeg shell in ground nutmeg. To the best of our knowledge, besides conventional microscopic methods [26], the detection of shell material in nutmeg has only been reported using hyperspectral imaging [14]. However, this study has been limited due to a small number of samples. Additionally, we observed that the coloring of nutmeg can change significantly (e.g., darken) during storage, which could affect the suitability of this method.

Fourier transform near-infrared (FT-NIR) spectroscopy is an extremely versatile and powerful method to verify the authenticity of food raw materials. It has been applied for a wide range of foodstuffs, for example, regarding the geographical or botanical origin of crops [17,27,28,29,30,31,32,33,34,35,36,37] or for the characterization of different sensory properties [38,39]. Hence, NIR spectrometers are established in many industrial routine laboratories for different food-related analyses, e.g., the quantitation of the moisture, protein, fat, and sugar content, to verify the composition of food ingredients, or as a fingerprinting technique [31,40]. The suitability of FT-NIR spectroscopy for process control can be attributed to the fact that the method is fast, reliable, easy to use, less expensive, non-destructive, and does not require hazardous chemicals [41].

NIR spectroscopy has been used to estimate quality attributes and to detect adulterations in various herbs and spices, for example, to detect cornstarch in onion powder [42], cumin [43], or turmeric powder [44]. Furthermore, many NIR-based methods have been developed to identify various falsifications (e.g., papaya seeds, black pepper husk, defatted spent) in black pepper [43,45,46,47]. It has already been demonstrated that NIR spectroscopy is suitable to detect adulteration of ground nutmeg with cumin, monosodium glutamate, soil, roasted coffee husks, and wood sawdust [48].

Therefore, the objective of this study was to develop a powerful, yet simple and routinely applicable FT-NIR-based method for the determination of seed shell content in ground nutmeg.

## 2. Materials and Methods

### 2.1. Sample Acquisition

A total of 36 authentic nutmeg samples and 10 nutmeg shell samples produced between 2015 and 2021 were analyzed (see Supplementary Materials Table S1 for detailed sample information). All samples originated from Indonesia and were acquired as whole nutmeg directly from the producers and exporters. The shell material was acquired in ground state sieved under 0.25 mm.

### 2.2. Sample Preparation

The whole nutmeg samples were industrially processed in Hamburg (Germany), including homogeneous grinding and sieving under a diameter of 0.4 mm. After processing, the samples were stored in airtight bags (Anton Debatin GmbH, Bruchsal, Germany) at room temperature for up to seven years until analysis, which was performed in 2021/2022.

Prior to FT-NIR analysis, a total of 1.50 g (±0.1 g) of the ground material was weighed into closed glass vials (52.0 mm × 22 mm × 1.2 mm, Nipro Diagnostics Germany GmbH, Ratingen, Germany) at 22 °C (± 2 °C). 

All 36 authentic nutmeg samples and the 10 shell samples were analyzed in pure form as well as in mixtures. To produce the mixtures, random combinations of nutmeg and shell samples were chosen with the following constraints. At least 7 different mixtures for each nutmeg sample and 35 mixtures for the shell samples were produced. Each ground nutmeg sample was mixed with 3% and 7% of ground shell material, while at all other percentages (1–10% in 1% steps and 10–50% in 10% steps), 15–23 mixtures each were analyzed. In total, 307 mixtures were obtained (230 mixed samples ranging from 1–10% shell content and 77 mixtures with shell percentage of >10%).

### 2.3. Spectra Acquisition

The samples and mixtures were analyzed in glass vials using an FT-NIR spectrometer with an integrating sphere (TANGO, Bruker Optics GmbH & Co. KG, Ettlingen, Germany). The spectra were acquired in reflectance mode with 50 scans per spectrum and a resolution of 4 cm^−1^ in a wavenumber range of 11,550–3950 cm^−1^. Data acquisition was carried out via OPUS 7.5.18 software (Bruker Optics GmbH & Co. KG, Ettlingen, Germany). All samples were measured at room temperature (22 °C ± 2 °C). Five spectra were acquired per sample/mixture. The glass vial was shaken thoroughly between the measurements.

### 2.4. Spectra Pre-Processing

**Figure 1:**
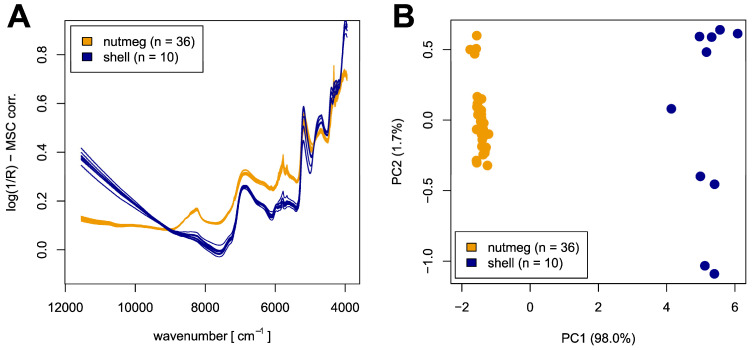
Overview of authentic nutmeg and nutmeg shell samples; (**A**) NIR spectra (MSC-corrected); (**B**) PCA scores plot after MSC correction including first and second principal component.

**Figure 2:**
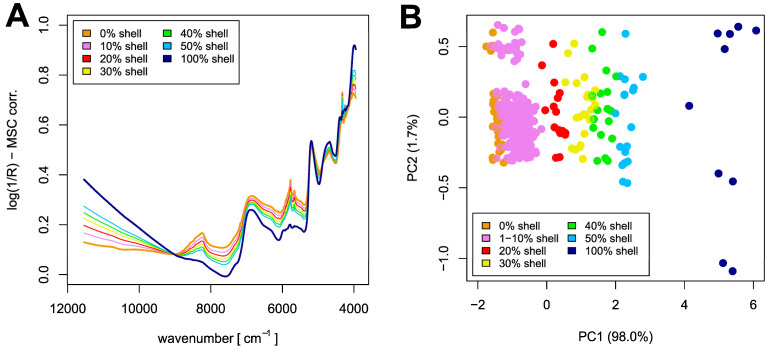
(**A**) Average spectra of nutmeg, nutmeg shell, and different mixtures of nutmeg with 10%, 20%, 30%, 40%, or 50% nutmeg shell proportion after MSC correction, bold type: spectrum of pure nutmeg and pure shell.; (**B**) PCA scores plot of nutmeg, nutmeg shell samples, and different mixtures of nutmeg with 1–50% nutmeg shell content (variances based on pure nutmeg and shell samples).

Data pre-treatment was carried out using R (version 3.6.1) including the package prospectr [49,50]. First, the five technical replicates per sample/mixture were averaged. Next, multiplicative scatter correction (MSC) was used in order to reduce additive and multiplicative scattering effects [51,52]. The mentioned scattering effects led to differences in the spectra caused by inhomogeneous physical effects and not—as desired—by shell content. As a reference spectrum for MSC correction, the average spectrum of all pure nutmeg and shell samples was used (see Figure 1 and Figure 2). In contrast, for the support vector machine (SVM) model, the average spectrum was calculated using the training samples only. The same spectrum was also used for processing of the test data to ensure that no information from the test set was used for the predictions. In addition to MSC correction, further pre-processing methods were tested, including standard normal variate (SNV), detrend combined with SNV, and MSC correction combined with a first derivative (smoothing: moving average with filter length = 101).

### 2.5. Multivariate Data Analysis

All statistical analysis was carried out using R (version 3.6.1) including the packages e1071, networkD3, and pls [49,53,54,55]. After pre-processing, principal component analysis (PCA) was performed with the prcomp function from the package stats with default parameters [49,56,57]. To allow for a better comparability, the same PCA transformation as for pure nutmeg and shell samples (Figure 1B) was used for the plot including all mixed samples (Figure 2B).

A linear SVM or PLS model was applied for the prediction of up to 10% shell content in ground nutmeg samples with 2-fold cross-validation. To this end, only mixtures with a nutmeg shell content of up to 10% were considered. As a training set, half of the nutmeg and half of the nutmeg shell samples were used, applying randomly chosen partitioning of the dataset. Furthermore, all mixtures, including only the chosen nutmeg and shell samples, were added into the training set. The remaining half of nutmeg, shell, and corresponding mixed samples were used as a test set. Training and test sets were switched for the second cross-validation loop. Since default parameters for the linear SVM and PLS were used, a nested cross-validation was not required. In this way, approximately one quarter of the mixed samples were integrated in the training and test set, respectively. This approach was repeated 100 times, leading to an approximate 50-fold prediction of each mixed sample. 

In a second step, an ensemble classifier was built by combining the classifier trained during the 100 cross-validation repetitions. The predicted shell content was defined as the averages of the approximately 50 predictions per mixed sample. Due to the design described above, no information about the predicted nutmeg sample or associated shell samples was used during classifier development in line with common machine learning practice [58].

## 3. Results

### 3.1. Data Set and Spectra Interpretation

An overview of the dataset used in this work is given in Figure 1. The spectra plot shown in Figure 1A illustrates the two groups of NIR spectra—of ground nutmeg and nutmeg shell—with distinct differences concerning intensity and position of characteristic peaks. In addition, the spectra of nutmeg samples seem to be more homogenous than those of the nutmeg shell. Since complex matrices like nutmeg inevitably cause overlapping peaks in the near-infrared region, an exact peak assignment is not possible. Nevertheless, the bands can be correlated with different substance classes.

The aliphatic hydrocarbon part of the lipids absorbs at different wavenumbers. At about 8260 cm^−1^, the second overtone of the C-H stretching in methylene is located, while the absorptions at about 5780 cm^−1^ and 5665 cm^−1^ are caused by the first overtone of the C-H stretching in methylene and that of the symmetric C-H_2_ bond vibrations, respectively. Furthermore, the second overtones of the C-H bending and C-H_2_ bending are located at about 4330 cm^−1^ and 4250 cm^−1^, respectively. The intensity of all lipid-associated bands is higher in the spectra of nutmeg than in those of the shell samples, which is consistent with the higher lipid content in nutmeg [59,60].

In addition to the lipid-associated bands, further characteristic absorptions can be correlated, in particular with the carbohydrate content: At about 6890 cm^−1^, the O-H stretch (first overtone) as well as the C=O stretch (third overtone) absorb, while the combination vibrations of O-H/C-O stretch occur at about 4400 cm^−1^. Additionally, the band at about 4000 cm^−1^ is caused by the combination of C-H stretch and C-C stretch vibrations. The carbohydrate-associated bands show a higher intensity in the spectra of the shell samples, indicating that the carbohydrate content in the shell is higher than in nutmeg [59,60].

Figure 1B shows the PCA scores plot of all nutmeg and shell samples used. Based on the first principal component, a complete segregation of both sample groups is visible. Furthermore, nearly the complete variance (98.0%) is described by the first principal component. Thus, the PCA plot suggests that a clear linear separation between both groups is possible based on the information described by PC1. As already observed in the spectra plot (Figure 1A), the nutmeg shell samples show a higher variance than the nutmeg samples. This variance seems to be contained, in particular, within the second principal component (1.7%). The PCA scores plots resulting from different pre-treatment methods as described in the section spectra pre-processing were observed to be quite similar (see Supplementary Material for further PCA scores and loadings plots).

### 3.2. Mixed Samples

The spectra plot displayed in Figure 2A shows the average spectra of the analyzed mixture samples. As expected, these spectra are located in regular intervals in between the average spectra of nutmeg and nutmeg shell samples, with the 10% shell sample spectra being closest to the pure nutmeg spectra and the 50% mixtures closest to those of the nutmeg shells. This already suggests the suitability of NIR spectroscopy for the quantitation of the nutmeg shell content in ground nutmeg samples. Afterwards, the PCA-transformed data of mixed samples were computed, which is displayed in Figure 2B. The PCA scores plot shows a clear grouping of the different mixtures horizontally aligned with the lowest shell content on the left side of the plot at low PC1 values and the mixtures with 50% nutmeg shell content at higher PC1 values and right in the middle of pure nutmeg and shell samples. Each group of mixtures with a specific nutmeg shell percentage is vertically spread over the plot. The variance of the groups of mixtures is comparable to that of the nutmeg samples. 

Moreover, the PCA scores plot displays that all mixed samples with a shell content of >10% can be easily separated from the pure nutmeg samples. For this reason, and to assess the capacity of the machine learning model to recognize adulteration with low percentages, only mixtures with a shell content of 1–10% were included in the statistical model.

### 3.3. Statistical Analysis for Prediction of Nutmeg Shell Percentage

**Figure 3:**
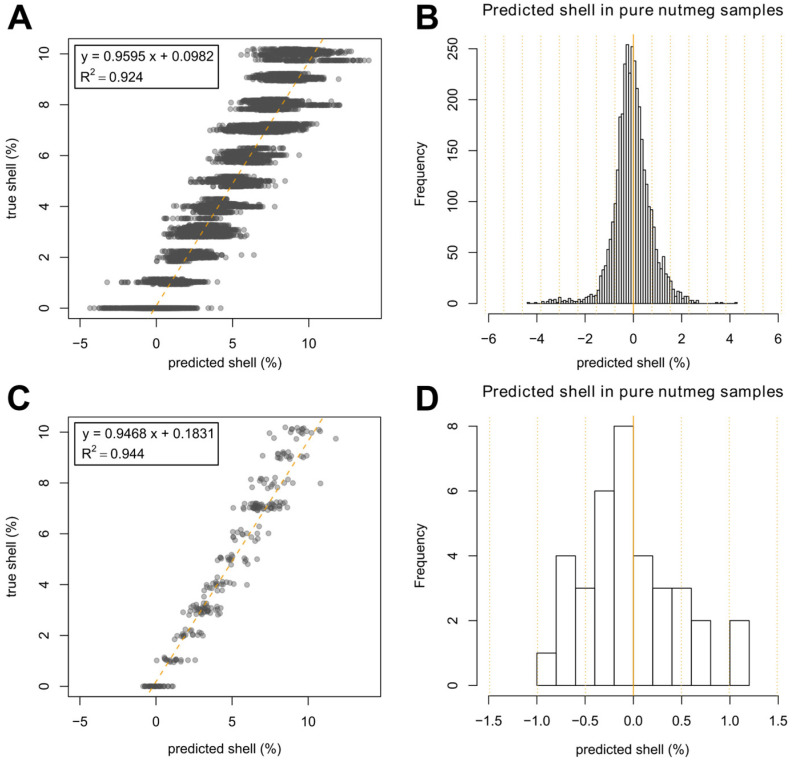
(**A**) Scatter plot of predicted vs. true nutmeg shell percentage in nutmeg samples based on 100-fold repeated SVM prediction; (**B**) Histogram showing the frequency of predicted shell percentages in pure nutmeg samples taken from the scatter plot (**A**); (**C**) Scatter plot of predicted vs. true shell percentage in nutmeg samples based on the average of 100-fold repeated SVM prediction; (**D**) Histogram showing the frequency of predicted shell percentage in pure nutmeg samples taken from the scatter plot (**C**). The orange dotted lines of both histograms visualize the standard deviation (σ).

To develop a model for prediction of the nutmeg shell content, an SVM algorithm was applied using two-fold cross-validation. In the first step, half of the nutmeg samples and half of the nutmeg shell samples were used for training of the model. Furthermore, all corresponding mixed samples containing both chosen nutmeg and shell samples were included in the training set. Accordingly, the remaining nutmeg and shell samples as well as the corresponding mixed samples were used as test set. For the second cross-validation loop, the training and test sets were switched. This procedure was repeated 100 times to determine the deviation between single predictions. In this way, each mixture was predicted 50 times on average. All these predictions are displayed in the scatter plot in Figure 3A. A clear linear relationship of true and predicted nutmeg shell content can be observed, with the coefficient of determination (*R*^2^) in the range of 1–10% shell content being 0.924. In addition, the root mean squared error of prediction (RMSEP) in this range was determined with 0.93%. In comparison, a PLS model achieved a similar, but slightly lower *R*^2^ of 0.914 (cf. Figure S6).

The predicted shell percentages in pure nutmeg samples are represented in Figure 3B as a histogram of all single prediction results of the shell content (100 predictions per sample) combined with the respective frequency. A Gaussian distribution around a nutmeg shell proportion of 0% is observed with a maximum of 4.2 percentage points. The standard deviation (σ) of this distribution is 0.77% (orange dotted line). As an example, a prediction result of 1% nutmeg shell corresponds to a z-score of 1.3 σ or, in other words, to an area under the normal curve of 90.320%. In detail, this indicates that for a pure nutmeg sample, the likelihood that a prediction score of >1% is obtained with the applied statistical model is 9.680% based on a normal distribution (*p* = 0.0968).

To make optimal use of the available data and to get a more distinct view of the results, we developed an ensemble classifier (see section Multivariate Data Analysis). To this end, the arithmetic average of all predictions was determined for each single mixture. The result is presented as a scatter plot in Figure 3C. The variance of all mixed samples, i.e., the deviation of predicted vs. true shell content, was further reduced (*R*^2^ = 0.944) in comparison to the scatter plot with all single predictions shown in Figure 3A. Likewise, the RMSEP was decreased to 0.75%.

Accordingly, a histogram showing the frequency of the shell content predicted in all pure nutmeg samples is displayed in Figure 3D as an average of all 100 single predictions for each nutmeg sample. The average prediction was observed to be below 1.5% for all pure nutmeg samples included in this study—with a maximum of 1.12%. In addition to a decreased maximum prediction score, the standard deviation was reduced to σ = 0.50%. Consequently, a predicted nutmeg shell content of 1% corresponds to a z-score of 2 σ or to an area under the normal curve of 97.725%. As a result, applying the presented model with all combined predictions, the probability that a pure nutmeg sample is predicted with a shell content of >1% is 2.275% (*p* = 0.02275). 

To assess the performance of the developed method to detect an adulterated nutmeg sample, the limit of detection (LOD) should be determined. A widely used approach in analytical method validation for determining the LOD and the limit of quantitation (LOQ) is based on the 10-fold measurement of a blank sample. LOD and LOQ are afterwards derived from the standard deviation of this 10-fold blank analysis with three-fold standard deviation being considered as the LOD and nine-fold standard deviation as the LOQ [61].

Transferring this approach to the problem of detecting a nutmeg sample adulterated with nutmeg shell, measurements of pure authentic nutmeg samples can be considered as “blank samples” since no nutmeg shell is contained. The predictions of the pure nutmeg samples are the results of those “blank” measurements. Due to the complexity of the underlying model, a resampling technique was applied. Based on 100 repeats, the standard deviation (σ) of the averaged predictions as displayed in the histogram (Figure 3D) was 0.5% nutmeg shell content. Applying this value, the resulting 3 σ of our method is 1.5%, and 9 σ is 4.5% nutmeg shell. 

**Figure 4:**
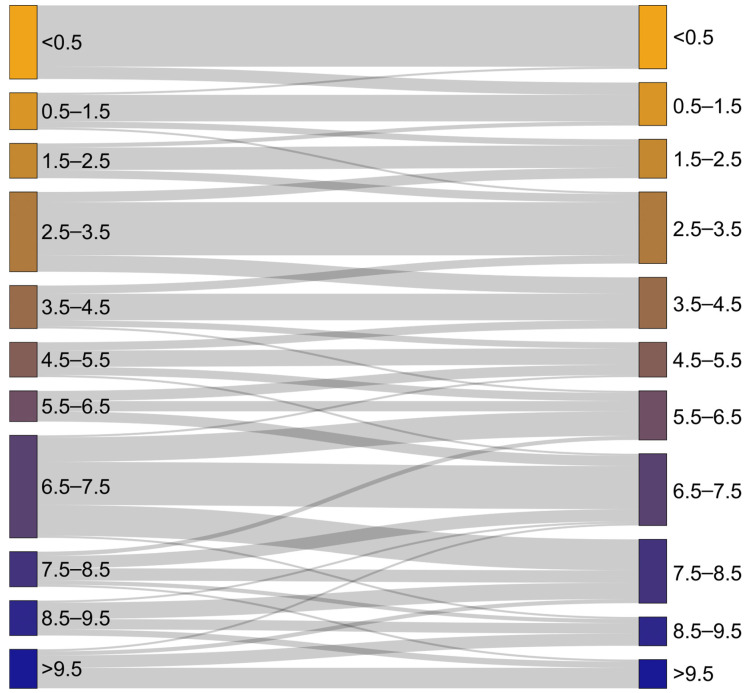
Sankey plot showing the relationship between true (**left**) and predicted shell percentage (**right**) based on the average predictions shown in Figure 3C,D.

The Sankey plot shown in Figure 4 allows for an alternative visual comparison of true vs. predicted nutmeg shell content. On the left side, all pure nutmeg samples as well as the mixed samples with nutmeg shell ≤10% are represented with their true nutmeg shell percentage. In contrast, the assignment according to the predicted shell content is shown on the right side. This type of chart provides traceability of the prediction result between groups of samples with the same nutmeg shell content. For pure nutmeg samples, the majority of samples is also predicted as “pure” in terms of a shell percentage of <0.5%. Only a small part of those samples is classified into the group of around 1% nutmeg shell content. In general, the largest proportion of samples is equivalent between true and predicted nutmeg samples, while smaller parts of each group are classified into the neighboring group of smaller or larger shell percentage. Only exceptionally, the classification takes place in the second or third neighboring group, meaning that there is a discrepancy of true vs. predicted shell content of 1–3% or 2–4%, respectively. 

## 4. Discussion

The NIR spectra plots shown above (Figure 1A) suggest that the two groups of interest—nutmeg and nutmeg shell—can easily be distinguished based on the acquired spectral data. This hypothesis is supported by the PCA scores plot showing full separation of both groups (Figure 1B). Furthermore, the scores plot already displays that a linear separation is possible based on the information represented by PC1. Moreover, when the mixed samples were added, a sequential arrangement of the mixtures following arithmetic logic from pure nutmeg with no added shell up to 100% nutmeg shell content was observed (Figure 2B). Again, separation of the groups with different shell percentages could be carried out linearly based on the information included in PC1. This observation was confirmed by the fact that a linear SVM model was well-suited for nutmeg shell prediction. It enabled detection of adulteration with nutmeg shell of ≥1.5% in a ground nutmeg sample. In addition, a certain quantitation of the mentioned adulteration is possible from 4.5%.

However, the explained approach of determining the LOD and the LOQ is only partly applicable to the analytical and statistical problem discussed in this work. The presented method is not an analytical measurement in terms of the analysis of a specific substance but a prediction of the shell content in ground nutmeg samples based on a machine learning model. In contrast to a blank of a chemical substance, which is measured repeatedly, the pure nutmeg samples contain some natural variation themselves, due, for example, to changing climatic conditions (e.g., temperature and precipitation). This variance was considered by including not only one but 36 different nutmeg samples (that were processed between 2015 and 2021). In addition, variance due to different storage times of the samples was also covered, as all samples were analyzed in 2021/2022—after storage times of up to seven years. Furthermore, the variance caused by different geographic origin was minimized in this work, as all samples originated in Indonesia, which reflects the country’s importance as the biggest nutmeg producer worldwide [4]. As a result of the discussed aspects, the values calculated following the concepts of LOD and LOQ explained above are considered as estimated limits since no standard procedure has been defined so far for a machine learning approach like the one presented in this study [61].

It is assumed that the amount of nutmeg shell added in adulterations is in the range of up to 10%, which would be an order of magnitude relevant to increased profits but without an obvious change of the sensory properties of nutmeg, which is therefore not easily detectable (compare Figure S1). The method presented in this work combined with the considerations of the LOD and the LOQ is well suited to detect an adulteration in this range. Even though a quantitation of nutmeg shell content is not possible below 4.5% with a high degree of confidence, the method allows for reliable detection of an adulteration even at very low levels of added nutmeg shell—with an estimated LOD of 1.5%. This allows for robust detection of most samples with a shell content exceeding the accepted content of extraneous matter of 0.5% given in the Codex Alimentarius for whole and broken nutmeg where food fraud is suspected [16].

So far, the commonly used approach to detect unwanted parts in nutmeg is microscopy, as it is an easy analysis to perform, and many laboratories are traditionally equipped with a light microscope. However, this analysis often provides inaccurate results with very high variations between replicate tests. Furthermore, some procedures include a sample preparation step—such as de-fatting—prior to analysis, leading to a time-consuming method [14]. Moreover, this type of analysis involves a subjective component, as the results are less measurable but rather based on human decisions made after comparing microscopic images with reference materials, and well-trained personnel are required. These limitations, a time-consuming analysis with subjectively shaped decisions, can be effectively eliminated by using NIR measurements combined with the prediction model described.

Another method for authentication of nutmeg concerning the adulteration with nutmeg shell was described by Kiani et al. [14]. In this work, 15 authentic nutmeg samples and only one shell sample were included. Using hyperspectral imaging and an artificial neural network model, adulterations in nutmeg were predicted. A result, <5% adulteration was classified as “similar to authentic”, meaning that adulterations below 5% could not be certainly detected, which is much higher than the LOD of 1.5% presented in this work. In addition, the coefficient of determination for the regression of predicted vs. true adulteration was 0.916, while the *R*^2^ was 0.944 in this study in the range of 0–10% shell content. This underlines that the prediction of the nutmeg shell content in nutmeg provided by the analysis method presented in our study is more precise than previously described methods.

## 5. Conclusions

In this study, it was shown that FT-NIR spectroscopy in combination with a support vector regression model is suitable to detect and quantify a possible adulteration of ground nutmeg with nutmeg shell powder. By analyzing 230 mixtures of Indonesian nutmeg and shell samples with up to 10% shell content, a model that enables the prediction of the shell content with a high prediction accuracy—a *R*^2^ of 0.944 and a RMSEP of 0.75%—was developed. The transferability to nutmeg of other geographical origins would still have to be validated. NIR spectroscopy offers great potential for the application in at-line routine analysis, since it is an easy, fast, and low-cost technology that is already established in numerous laboratories and many industrial processes, such as incoming goods control [62,63]. In addition, for the NIR analysis of ground nutmeg, no further sample preparation is required. The samples are simply placed in glass vials, which are directly placed on top of an NIR device [43,46]. Thus, for the first time, our method enables the detection of adulteration of as low as 1.5% nutmeg shell content in less than ten minutes.

## Data Availability

The code and data are available upon reasonable request from the corresponding author for non-commercial use.

## Supplementary Materials

The following supporting information can be downloaded at https://www.mdpi.com/article/10.3390/foods12152939/s1: Table S1: Overview of all analyzed nutmeg and shell samples; Figure S1: Photograph of pure nutmeg and nutmeg shell sample as well as mixtures with 10% and 20% shell content; Figure S2: PCA loadings plot based on MSC corrected data; Figure S3: NIR spectra of nutmeg and nutmeg shell applying SNV/detrend and SNV/MSC correction combined with first derivative; Figure S4: PCA scores plots based on SNV treated data/detrend and SNV treated data/MSC correction combined with first derivative; Figure S5: PCA loadings plots based on SNV treated data/detrend and SNV treated data/MSC correction combined with first derivative; Figure S6: Scatter plot of predicted vs. true nutmeg shell percentage in nutmeg samples based on 100-fold repeated PLS prediction and based on the average of 100-fold repeated PLS prediction.
