# Supplementary material for: Rapid Determination of Nutmeg Shell Content in Ground Nutmeg Using FT-NIR Spectroscopy and Machine Learning
Source: Foods. 2023 Aug 2;12(15):2939. doi: 10.3390/foods12152939 (PMC10418458; doi:10.3390/foods12152939)
Supplement: Supplementary file 1 [file foods-12-02939-s001.zip › foods-2508313-supplementary.pdf]

Rapid Determination of Nutmeg Shell Content in Ground Nutmeg using FT-NIR Spectroscopy and Machine Learning

Alissa Drees [1,†], Bernadette Bockmayr[2,†], Michael Bockmayr[3,4] and Markus Fischer [1,5,*]

[1] Hamburg School of Food Science, Institute of Food Chemistry, University of Hamburg, Grindelallee 117, 20146 Hamburg, Germany; alissa.drees@uni-hamburg.de, markus.fischer@uni-hamburg.de
[2] Husarich GmbH, Peutestraße 53 D, 20539 Hamburg, Germany; b.bockmayr@husarich.com
[3] Department of Pediatric Hematology and Oncology, University Medical Center Hamburg-Eppendorf, Hamburg, Germany; m.bockmayr@uke.de
[4] Research Institute Children's Cancer Center Hamburg, Germany; m.bockmayr@uke.de
[5] Center for Hybrid Nanostructures (CHyN), Department of Physics, University of Hamburg, Luruper Chaussee 149, 22761 Hamburg, Germany; markus.fischer@uni-hamburg.de
* Correspondence: markus.fischer@uni-hamburg.de; Tel.: +49 40 42838-4359
† These authors contributed equally to this work.

# - Supplementary material -

Table S1: Overview of all analyzed nutmeg and shell samples with corresponding harvest year. All samples were harvested in Indonesia.

Sample ID	Type	Production year	Sample ID	Type	Production year
1	Nutmeg	2015	24	Nutmeg	2021
2	Nutmeg	2015	25	Nutmeg	2021
3	Nutmeg	2016	26	Nutmeg	2021
4	Nutmeg	2016	27	Nutmeg	2021
5	Nutmeg	2016	28	Nutmeg	2021
6	Nutmeg	2016	29	Nutmeg	2021
7	Nutmeg	2017	30	Nutmeg	2021
8	Nutmeg	2017	31	Nutmeg	2021
9	Nutmeg	2017	32	Nutmeg	2021
10	Nutmeg	2017	33	Nutmeg	2021
11	Nutmeg	2017	34	Nutmeg	2021
12	Nutmeg	2017	35	Nutmeg	2021
13	Nutmeg	2017	36	Nutmeg	2021
14	Nutmeg	2017	37	Nutmeg shell	2016
15	Nutmeg	2017	38	Nutmeg shell	2016
16	Nutmeg	2017	39	Nutmeg shell	2017
17	Nutmeg	2017	40	Nutmeg shell	2017
18	Nutmeg	2017	41	Nutmeg shell	2017
19	Nutmeg	2017	42	Nutmeg shell	2017
20	Nutmeg	2017	43	Nutmeg shell	2020
21	Nutmeg	2020	44	Nutmeg shell	2020
22	Nutmeg	2020	45	Nutmeg shell	2021
23	Nutmeg	2020	46	Nutmeg shell	2021

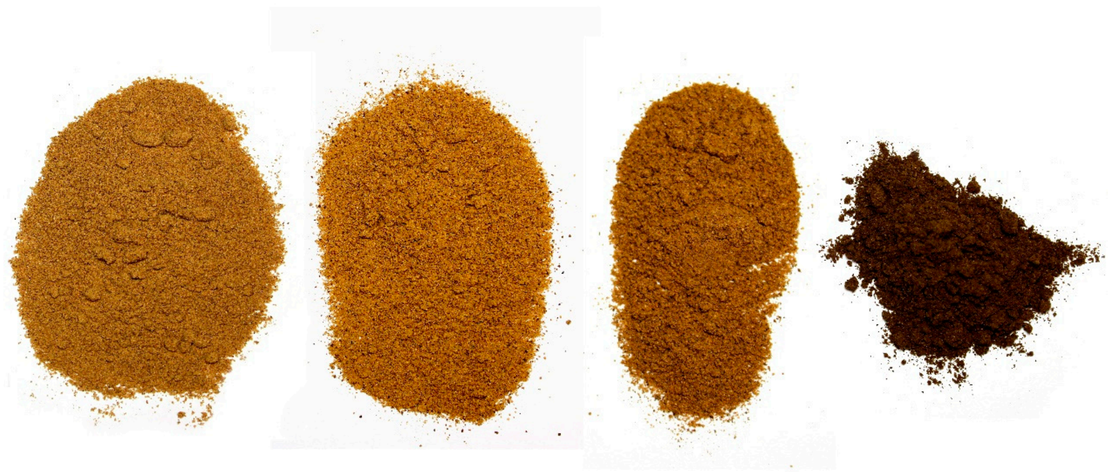

Figure S1: From left to right: Photographs of a nutmeg sample (ID 26), a mixture with 10% nutmeg shell, a mixture with 20% nutmeg shell, and a nutmeg shell sample (ID 39).

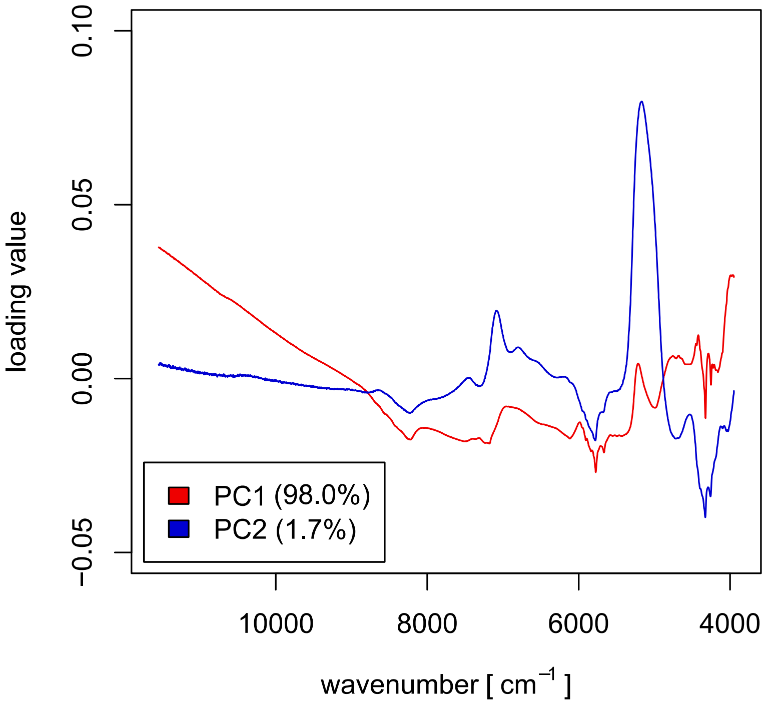

Figure S2: PCA loadings plot based on MSC corrected data.

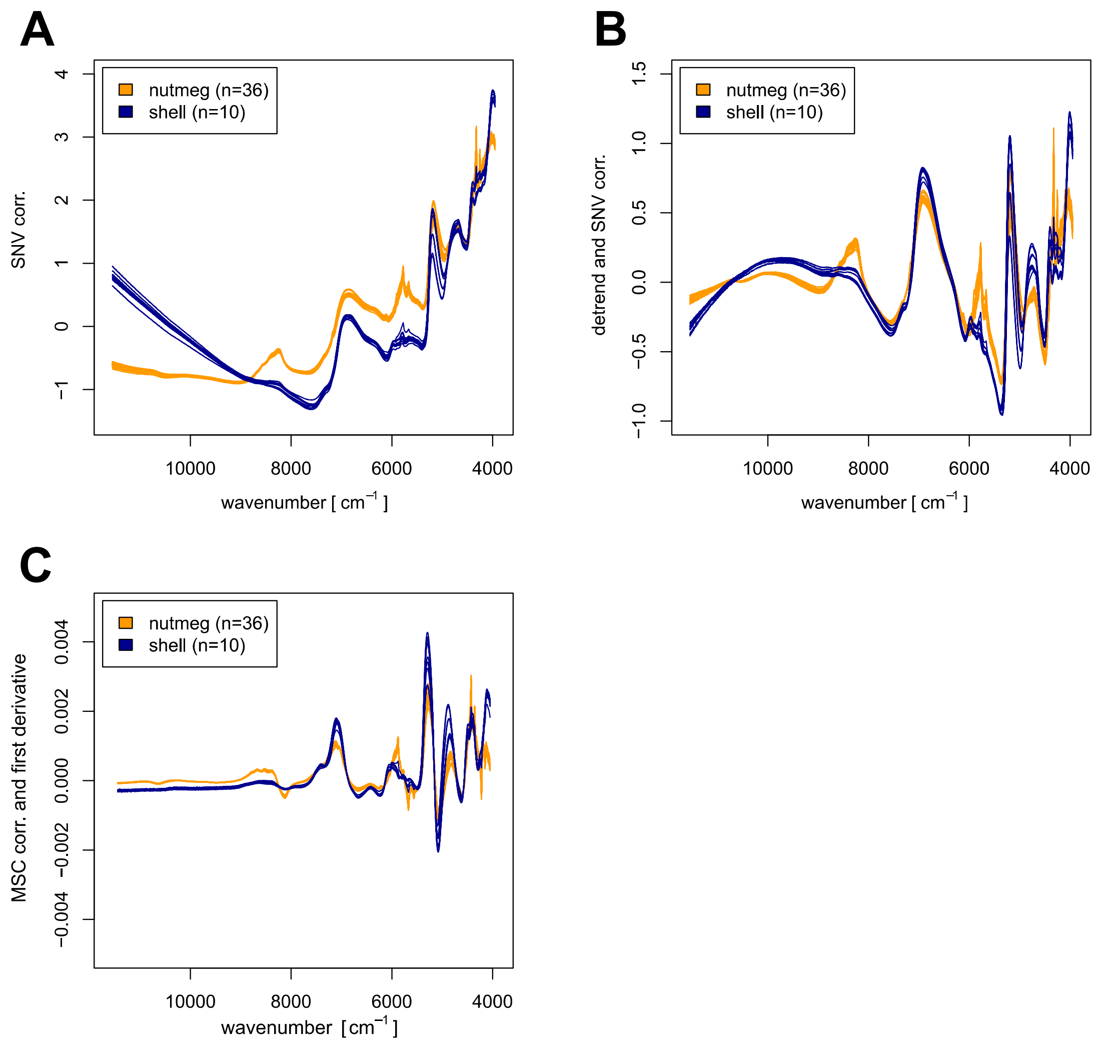

Figure S3: NIR spectra of nutmeg and nutmeg shell applying different pre-processing methods: **A** SNV treated data; **B** detrend and SNV treated data; **C** MSC correction combined with first derivative.

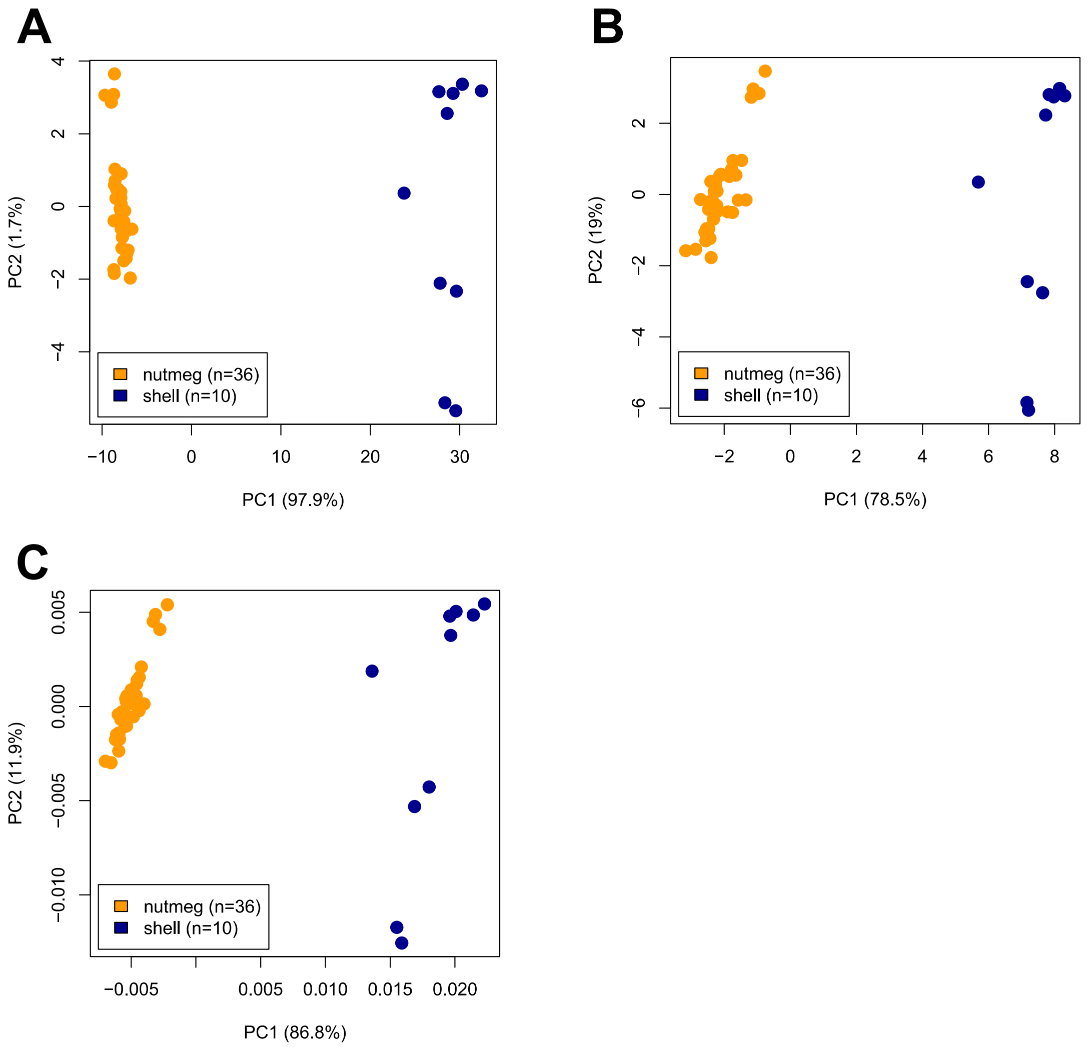

Figure S4: PCA scores plots based on different pre-processing methods: **A** SNV treated data; **B** detrend and SNV treated data; **C** MSC correction combined with first derivative.

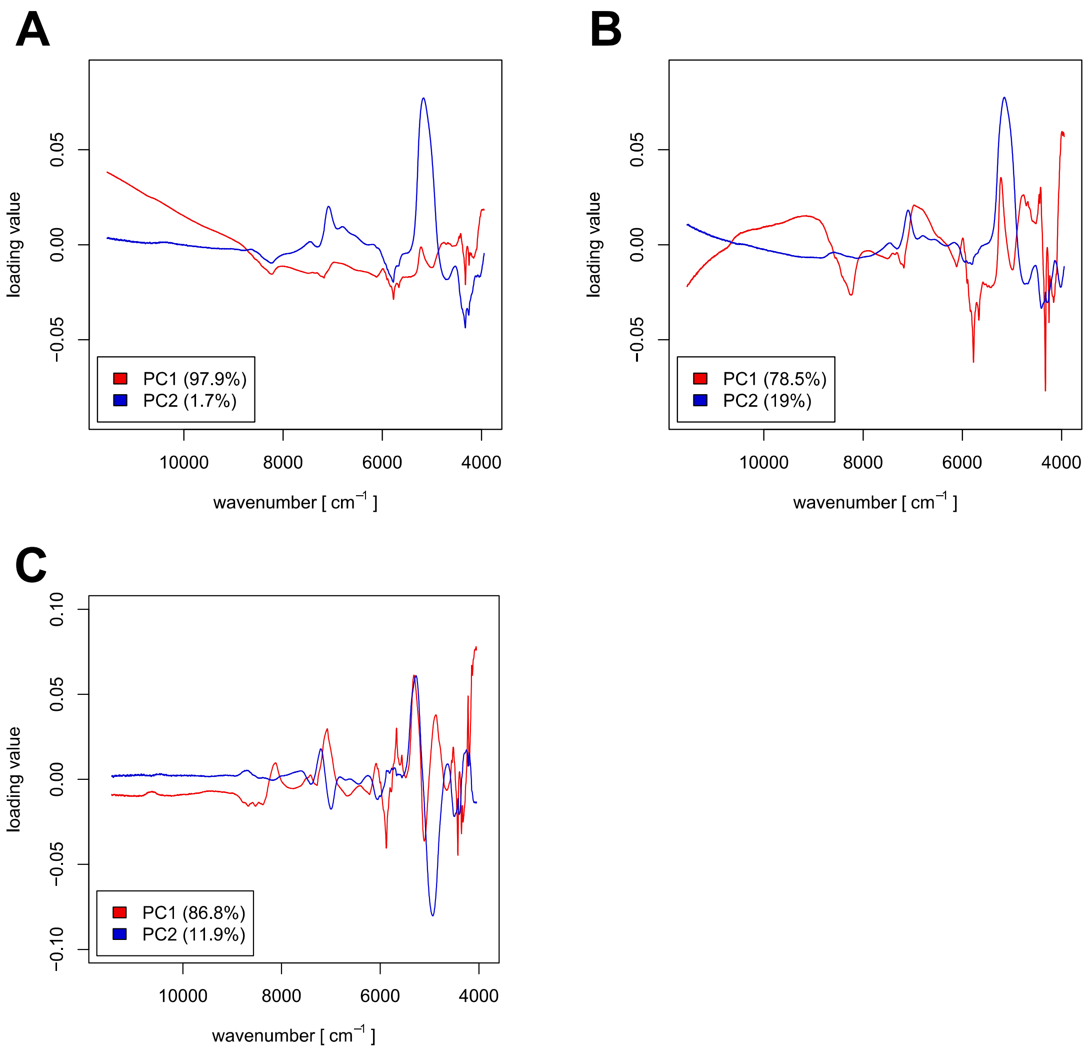

Figure S5: PCA loadings plots based on different pre-processing methods: **A** SNV treated data; **B** detrend and SNV treated data; **C** MSC correction combined with first derivative.

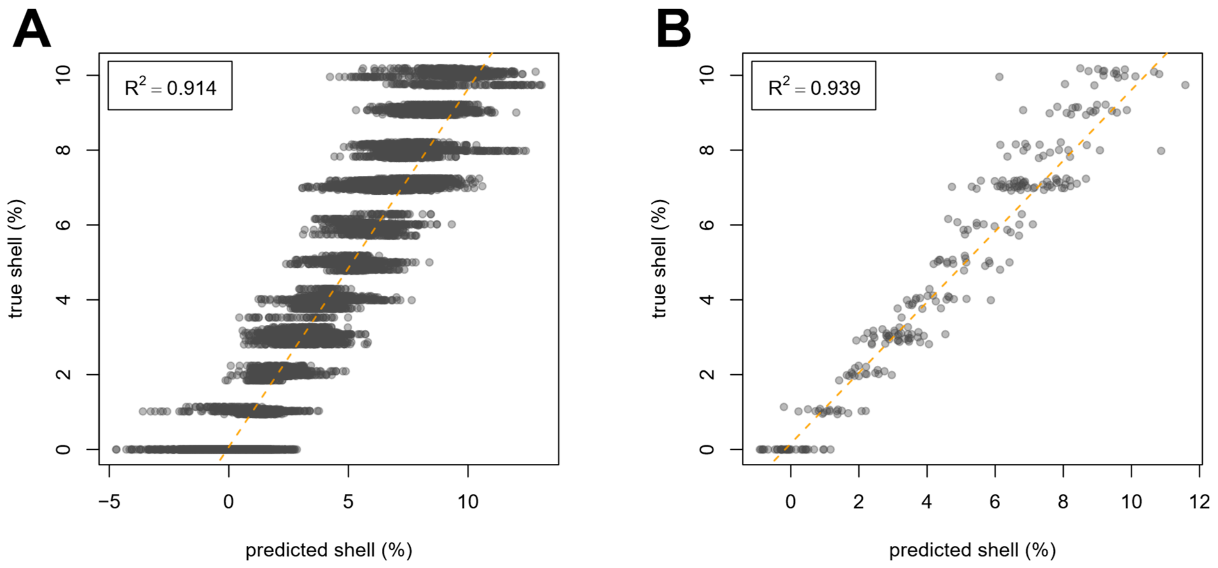

Figure S6: **A** Scatter plot of predicted vs. true nutmeg shell percentage in nutmeg samples based on 100-fold repeated PLS prediction; **B** Scatter plot of predicted vs. true shell percentage in nutmeg samples based on the average of 100-fold repeated PLS prediction.
